# Bioinspired, Mechano‐Regulated Interfaces for Rationally Designed, Dynamically Controlled Collection of Oil Spills from Water

**DOI:** 10.1002/gch2.201600014

**Published:** 2017-02-15

**Authors:** Yaoyao Li, Deyong Zhu, Stephan Handschuh‐Wang, Guanghui Lv, Jiahui Wang, Tianzhen Li, Cancheng Chen, Chuanxin He, Junmin Zhang, Yizhen Liu, Bo Yang, Xuechang Zhou

**Affiliations:** ^1^ College of Chemistry and Environmental Engineering Shenzhen University Shenzhen 518060 P. R. China

**Keywords:** elastomeric sponges, flexible devices, oil skimmers, surfaces and interfaces, water and oil separation

## Abstract

This study describes the fabrication of bioinspired mechano‐regulated interfaces (MRI) for the separation and collection of oil spills from water. The MRI consists of 3D‐interconnected, microporous structures of sponges made of ultrasoft elastomers (Ecoflex). To validate the MRI strategy, ecoflex sponges are first fabricated with a low‐cost sugar‐leaching method. This study then systematically investigates the absorption capacity (up to 1280% for chloroform) of the sponges to different oils and organic solvents. More importantly, the oil flux through the as‐made sponges is controlled by mechanical deformation, which increases up to ≈33‐fold by tensile strain applied to the sponge from 0 to 400%. On the basis of MRI, this study further demonstrates the application of ecoflex sponges in oil skimmers for selective collecting oil from water with high efficiency and durable recyclability. The as‐developed MRI strategy has opened a new path to allow rational design and dynamical control toward developing high performance devices for oil permeation and selective collection of oil spills from water.

In order to survive in nature, living systems undergo highly defined morphological evolution to adapt to environmental changes. For example, sponges,^1^ aquatic animals of the *phylum Porifera*, possess bodies full of pores, channels, and chambers, through which a water flow circulates, to bring food and oxygen, and to remove wastes. Therefore, sponges represent a biological paradigm for storage and release of water in a so‐called porous system. Possessing similar unique characteristics of natural ones, biomimetic systems have become more and more appealing.^2^, ^3^, ^4^, ^5^, ^6^, ^7^ Artificial sponges, which resemble the aforementioned porous and elastic animal sponges, have been utilized in various fields including clean‐up, packaging, thermal insulation, flame retardant, and soft electronics with materials, such as carbon, polyurethane, and polydimethylsiloxane (PDMS).^8^, ^9^, ^10^, ^11^, ^12^, ^13^, ^14^, ^15^, ^16^, ^17^, ^18^ The intensive investigations on such mechano‐regulated strategies adopted by the living systems and tremendous efforts on the biomimetic ones have opened a new path to the development of high‐performance devices in many fields, including energy, electronics, healthcare, and environment.^19^, ^20^, ^21^, ^22^

Oil pollution, on the other hand, has become, in recent years, a critical threat to the survival of living things and the sustainable development of the society. For example, petrochemical, textile, and food industries cause oil pollution in rivers, lakes, and oceans, including the precious drinking water.^23^, ^24^, ^25^ Therefore, an effective and cheap method to separate oil/water mixtures is of grave importance for society and industry. Conventional approaches to remove oil spills include absorption with micro‐/nanocomposites,^26^ in situ burning,^27^ vacuum suction,^28^ bioremediation,^29^ skimming,^30^ and flotation.^31^ However, these traditional methods suffer from their intrinsic limits of low efficiency and high operation cost, and some even suffer from secondary pollution. Here, advanced materials and approaches to separate the valuable resources, oils and water, are required.^26^, ^32^, ^33^, ^34^, ^35^, ^36^ In recent years, the so‐called smart materials arose and drew much attention in the scientific community. Smart materials are stimuli‐responsive materials, which react to various stimuli, such as illumination,^37^ pH,^38^, ^39^ temperature,^40^, ^41^, ^42^, ^43^, ^44^ chemical trigger,^45^, ^46^, ^47^ magnetic field,^48^, ^49^, ^50^ electric field,^51^ and deformation.^15^, ^16^, ^17^ For example, Xue et al.^41^ proposed a thermoresponsive block copolymer coated mesh to separate water and oil. Recently, a dual‐responsive material based on a pH and temperature responsive hydrogel‐coated mesh was suggested by Cao et al.^52^ While these new methods are elegant, they lack the applicability in challenges outside the laboratory. The responsive materials may have problems, such as slow reaction time, low recyclability, low efficiency, and poor usability to irregular and erratic interfaces. Therefore, new systems that function in complicated systems possess high chemical inertness and can operate under and after the mechanical stress is needed.

To address this need, we introduce a mechano‐regulated interface (MRI) for rationally designed, dynamically controlled oil permeation and the application in skimmers for collecting oil spills from water (**Figure** **1**). In the present MRI strategy, the oil permeability is regulated by either extension or compression of the MRI. The key novelty of this strategy is that the bioinspired, mechano‐regulated interface features a controlled permeability of oils, whereas the interface retains its impermeability toward water (Figure 1a). To validate this strategy, we fabricated the MRI with 3D‐interconnected, microporous sponges with Ecoflex. Notably, Ecoflex is hydrophobic and an ultrasoft elastomer with giant stretchability (up to 900% tensile strain). To characterize the liquid absorption performance of Ecoflex sponges, absorptivity tests were conducted for various organic solvents, ranging from polar solvents like ethanol to nonpolar solvents like hexane, and absorption and releasing cycles for paraffin oil were executed to signify the reusability of the sponge. What is more, to demonstrate the mechano‐regulated features on oil flux, the permeation rate of paraffin oil was controlled either by the thickness of the sponge or the mechanical strain applied to the sponge. As proof‐of‐concept, we successfully demonstrated the application of an MRI‐integrated skimmer to automatically collect oil spills from water (Figure 1b).

**Figure 1:**
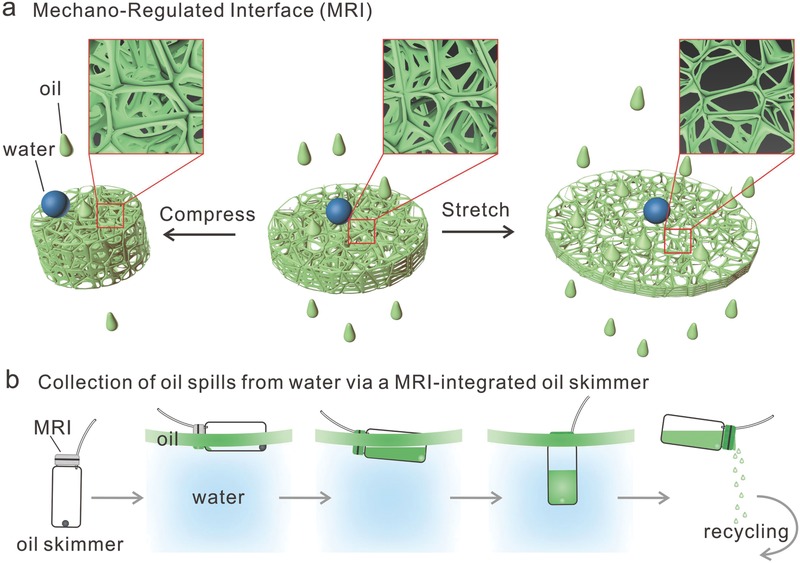
a) Schematic illustration of the bioinspired mechano‐regulated interfaces (MRIs) of hydrophobic and ultrasoft sponges made out of elastomer for dynamically controlled oil flux by stretching and compressing. b) Schematic illustration of the MRI‐integrated oil skimmer and its application in collecting oil spills from water.

To fabricate the MRI with Ecoflex, we applied a low‐cost and environmentally friendly method, the so‐called sugar leaching. Notably, the key feature of the MRI is that the interface consists of 3D‐interconnected, microporous structures of ultrasoft elastomer. Previously, we have applied such a sugar‐leaching method to fabricate PDMS sponges to construct 3D compressible and stretchable conductors.^19^ In the present study, we followed a similar procedure to fabricate Ecoflex sponges (Figure S1, Supporting Information). **Figure** **2**a shows an image of this 3D sugar template with the dimensions of 20 × 20 × 11 mm^3^ (*l*/*w*/*h*). The sugar crystals and the voidage between these crystals are clearly visible. After the template‐assisted fabrication, the as‐made sponge (Figure 2b) possesses dimensions similar to the template (17 × 17 × 9 mm^3^), of which the out layer has been removed to expose the sugar for dissolving. The porous structure of the sponge is visible by naked eye and is exemplified in the scanning electron microscopy (SEM) micrograph in Figure 2c. The as‐made sponge is highly flexible and exhibits a high elasticity (Figure 2d,e). After compression of the sponge, it will rapidly regain its former shape (Movie S1, Supporting Information). The wetting properties of the sponge were investigated by contact angle measurements and showed—with a water contact angle of 124° ± 2°, larger than that of the smooth Ecoflex patch (106° ± 5°), and a paraffin oil contact angle of ≤1°—the hydrophobic and superoleophilic properties of the sponge (Figure S2, Supporting Information). As shown in Figure 2f, water features a nearly round shape on the porous sponge, whereas the paraffin oil spreads on the sponge, while it is absorbed (Movie 2, Supporting Information). Such differences on wetting ability are attributed to the molecular and porous structures of the sponges, of which not only the roughness but also the air trapped within the micropores significantly increases.

**Figure 2:**
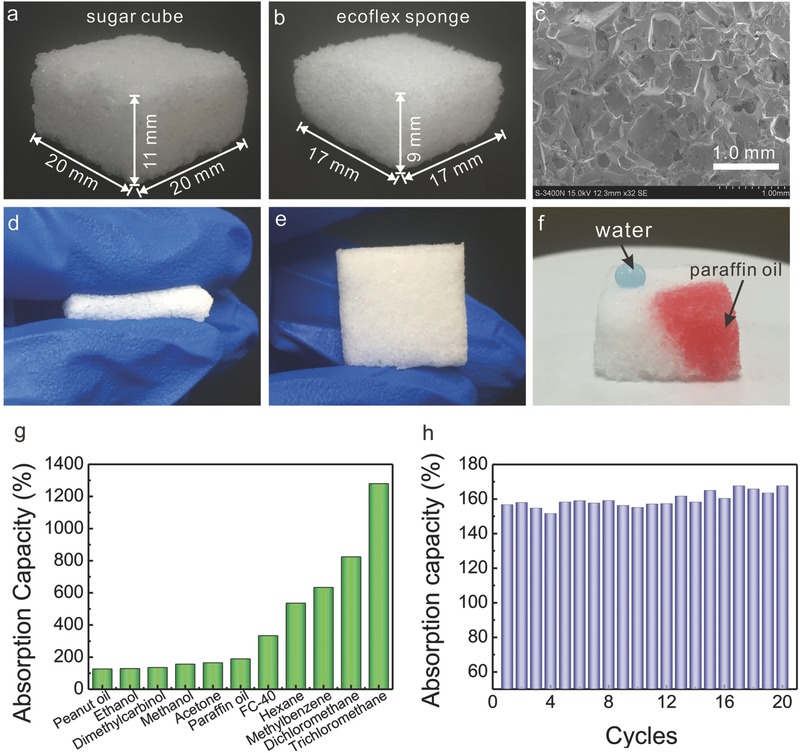
Digital images of a) a sugar cube and b) an as‐made sponge. c) SEM image of an Ecoflex sponge. Digital images of a sponge during d) compression and e) relaxing. f) Digital image of the water droplet (doped with blue dye) and paraffin oil (doped with red dye) deposited on an as‐made sponge. g) Absorption capacity of as‐made Ecoflex sponges for different organic solvents. h) Absorption capacity of paraffin oil at different loading cycles.

Importantly, the as‐made sponges exhibited significant absorption capability and reliable recyclability upon absorption to various organic compounds spanning from peanut oil to chloroform. A detailed description of the measurement procedure for the absorption capacity of the sponges is given in Equation (S1) in the Supporting Information. Indeed, the sponge exhibited absorption capacities ranging from 127% (peanut oil) to 1280% (chloroform) (Figure 2g). Very interestingly, the extreme high absorption capacity of chloroform and dichloromethane can be related to the high density of the solvents and their swelling property, which is in good agreement with previous studies of porous PDMS sponges.^15^ The nonpolar solvents, hexane and methylbenzene, have a relative large absorption capacity compared to peanut oil, paraffin oil, and FC‐40, which are attributed to the differences in swelling ability. Nevertheless, our Ecoflex sponge may possibly be utilized to selectively absorb both nonpolar and polar organic solvents. We thus successfully demonstrated the separation of water/paraffin oil and water/chloroform, as shown in Figure S3 and Movies S3 and S4 in the Supporting Information. More interestingly, enhanced durability and recyclability of the Ecoflex sponges during absorption and release of the oils and organic solvents were observed. Notably, the recyclability of the sponge and the recoverability of the oils and organic solvents are key factors for any practical application. In the present study, a loaded Ecoflex sponge releases readily its adsorbed oils and organic solvents by squeezing manually. The resulting graph in Figure 2h illustrates that the absorption capacity is virtually constant, perhaps showing a miniscule improvement during the advancement of the reusability experiment. Similar results were observed even for the organic solvent with high swelling property like chloroform (Figure S4, Supporting Information). The constant absorption capacity signifies the practicability of the composite as a durable, cheap, and environmentally friendly device for oil (or organic solvents) water separation.

More importantly, the oil permeation of the MRI made of Ecoflex sponges can be either tuned irreversible by the thickness of the sponge/sponge membrane or, as a specific feature of our sponge, reversible by a mechanical stress, which induces an increase in pore diameter. To determine the oil flux and validate our MRI strategy, we thus developed an oil skimmer (Figure 1b). Briefly, the oil skimmer comprises a glass bottle or glass vial, which is closed with the sponge, and a tube to release the pressure (Figure 1b). The pressure equalization is necessary as the oil is rapidly wetting the total surface of the polymeric sponge, thus blocking the air flow. The skimmer was filled with paraffin oil via the sponge lid. The flow rates were calculated by adapting an oil skimmer device (Equation (S2), Supporting Information).

Very interestingly, by varying the thickness of the sponge, the oil flux varied between 285 ± 69 g s^−1^ m^−2^ (thickness: 8.0 mm) and 1690 ± 73 g s^−1^ m^−2^ (thickness: 2.0 mm) at a tensile strain of 260 ± 40% (**Figure** **3**). The oil flux linearly decreased from 2 to 8 mm. This is explained by taking into account the linear increase of the distance for the oil to penetrate through. More importantly, the reversible adjustment of the oil flux was accomplished by varying the tensile stress between 0 and 400%, while the thickness was kept constant. The oil flux increased monotonically with increasing applied tensile stress from 0 to 400%. The increment of the tensile stress enlarges the pores of the sponge, which increases, in return, the oil flux.^53^ The relative incline was dependent on the thickness of the MRI. While a relative thin sample (1.7–4.7 mm) yielded a broad spectrum of oil flux rate, with an increase of up to 371–3343% of its flux rate, the spectrum of oil flux rate was narrowed by utilizing thicker samples, such as 7.4 mm (151%). As shown in **Figure** **4**b,c, a SEM micrograph of the sponge under a strain of ≈200% is shown, indicating the enlarged pores in comparison to the relaxed sponge (Figure 2c). The effect of pore enlargement on flux was also observed recently by Yan et al.^30^ for their oil skimmer device based on a 3D printed mesh. For comparison, compressing the MRI dramatically decreased the oil flux to 7 g s^−1^ m^−2^. This result verifies that the oil flux of the MRI indeed can be tuned by mechanical stretching or compressing.

**Figure 3:**
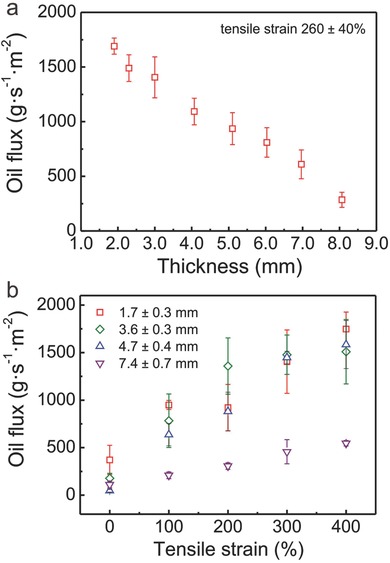
Oil flux a) versus the thickness of the MRI and b) versus the variation of the tensile strain for MRI sponges with different thicknesses.

**Figure 4:**
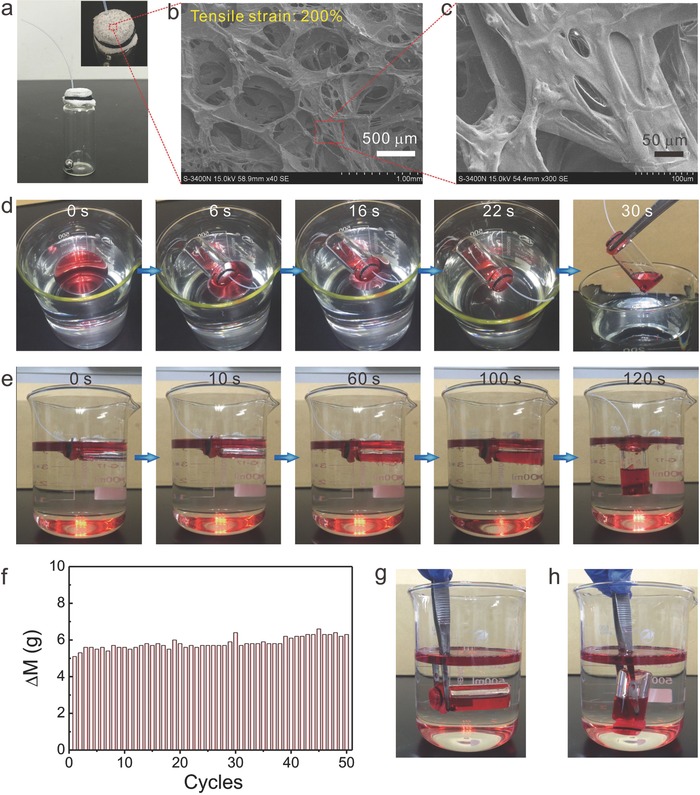
a) Photograph of an oil skimmer. SEM micrograph of the b) Ecoflex sponge lid and c) its magnification. d) Application of the oil skimmer (part (a)) to a spill of paraffin oil at the water/air interface, indicating the total removal of the spill. e) The application of the oil skimmer for a big paraffin spill at the water interface, indicating the uprising of the glass vial as it gets filled. f) Reusability test of the oil skimmer with paraffin oil for 50 cycles. g,h) Leak tightness test for tilting angles of 90° and 180° (total immersion into water).

Most importantly, the mechano‐regulated property on varying oil flux via mechanical deformation of Ecoflex sponges provides a new paradigm for the design of high‐performance oil skimmers. Notably, oil skimmers are one promising approach for cleaning up oil spills when compared with the strategy of oil‐absorbing and storing materials, which would become really heavy after absorption, time, and labor consuming to get rid of the oil. Skimming a specific oil/water mixture was reported by using a 3D‐printed skimmer, which overcame the previously mentioned restrictions.^54^ As proof‐of‐concept, in the present study, we demonstrated the application of the MRI made of Ecoflex sponge in an oil skimming device and show the reusability of the device (Figures 1b and 4d–h). The oil skimmer can remove a spill completely and the oil cannot escape from the oil skimmer, once it has been captured. Figure 4d shows the capturing of 5 mL of paraffin oil on water surface by the as‐made oil skimmer in 30 s. As illustrated in Figure 4e, the oil skimming process is rather fast. During the separation process, the oil skimmer fills with oil, which renders it heavier. After ≈100 s, the oil skimmer starts tilting due to the mass gain and in the subsequent 20 s the skimmer positions into an upright position (opening above), normal to the water/air interface (Movie 5, Supporting Information). The leak tightness of the device can be, in parts, ascribed to the 3D positioning of the skimming device. However, upon horizontal tilting in water or even pointing the lid down in water, no leakage was observed (Figure 4g,h). This attests the skimmer's high potential application to irregular and erratic interfaces, such as the open sea with its waves or even at storm conditions. In addition, the oil skimmer shows a high durability of usage (Figure 4f).

In conclusion, we have developed a bioinspired MRI strategy and its application in oil skimmers to capture oil spills from water. In this strategy, the MRI was made out of superhydrophobic and superoleophilic sponges from ultrasoft Ecoflex elastomer. Importantly, the Ecoflex sponges exhibit a high elasticity and mechanical durability upon stretching and compressing. Significantly, the sponges absorbed organic liquids, such as ethanol, toluene, paraffin oil, and chloroform, with an absorption capacity between 127 and 1280%. More interestingly, the oil flux through the MRI was regulated by strain applied to the sponge during the separation process, which increases up to ≈33‐fold by tensile strain applied to the sponge from 0 to 400%. A proof‐of‐concept application of a leakage‐free, highly selective oil skimming device is demonstrated by collecting paraffin oil from water. We envision that the oil skimming device can be readily employed to separate oil/water mixtures due to oil spills, especially at locations with highly erratic surfaces. However, not limited to the oil separation problems, the as‐developed strategy of mechano‐regulated interfaces should have implications on wettability control of surfaces, flexible electronic devices, soft healthcare devices, etc.

## Supporting information

As a service to our authors and readers, this journal provides supporting information supplied by the authors. Such materials are peer reviewed and may be re‐organized for online delivery, but are not copy‐edited or typeset. Technical support issues arising from supporting information (other than missing files) should be addressed to the authors.

Supplementary

Supplementary

Supplementary

Supplementary

Supplementary

Supplementary

## References

[1] J.‐M. Kornprobst , in Encyclopedia of Marine Natural Products, Vol. 2, Wiley‐VCH Verlag GmbH & Co. KGaA, Weinheim, Germany 2014, p. 521.

[2] A. S. Gladman , E. A. Matsumoto , R. G. Nuzzo , L. Mahadevan , J. A. Lewis , Nat. Mater. 2016, 15, 413. 26808461 10.1038/nmat4544

[3] L.‐B. Mao , H.‐L. Gao , H.‐B. Yao , L. Liu , H. Cölfen , G. Liu , S.‐M. Chen , S.‐K. Li , Y.‐X. Yan , Y.‐Y. Liu , S.‐H. Yu , Science 2016, 354, 107. 27540008 10.1126/science.aaf8991

[4] R. Schirhagl , C. Weder , J. Lei , C. Werner , H. M. Textor , Chem. Soc. Rev. 2016, 45, 234. 26750081 10.1039/c5cs90129d

[5] B. Wang , W. X. Liang , Z. G. Guo , W. M. Liu , Chem. Soc. Rev. 2015, 44, 336. 25311259 10.1039/c4cs00220b

[6] U. G. K. Wegst , H. Bai , E. Saiz , A. P. Tomsia , R. O. Ritchie , Nat. Mater. 2015, 14, 23. 25344782 10.1038/nmat4089

[7] H. Shahsavan , S. M. Salili , A. Jakli , B. X. Zhao , Adv. Mater. 2015, 27, 6828. 26418411 10.1002/adma.201503203

[8] V. Chabot , D. Higgins , A. P. Yu , X. C. Xiao , Z. W. Chen , J. J. Zhang , Energy Environ. Sci. 2014, 7, 1564.

[9] M. Langner , S. Agarwal , A. Baudler , U. Schroder , A. Greiner , Adv. Funct. Mater. 2015, 25, 6182.

[10] C. P. Ruan , K. L. Ai , X. B. Li , L. H. Lu , Angew. Chem., Int. Ed. 2014, 53, 5556. 10.1002/anie.201400775 24711147

[11] S. H. Jeong , S. Zhang , K. Hjort , J. Hilborn , Z. G. Wu , Adv. Mater. 2016, 28, 5830. 27167137 10.1002/adma.201505372

[12] L. Li , J. Zhang , Adv. Mater. Interfaces 2016, 3, 1600517.

[13] Y. Lim , M. C. Cha , J. Y. Chang , Sci. Rep. (UK) 2015, 5, 15957. 10.1038/srep15957 PMC4632124 26534834

[14] X. Wu , Y. Han , X. Zhang , Z. Zhou , C. Lu , Adv. Funct. Mater. 2016, 26, 6246.

[15] S. J. Choi , T. H. Kwon , H. Im , D. I. Moon , D. J. Baek , M. L. Seol , J. P. Duarte , Y. K. Choi , ACS Appl. Mater. Interfaces 2011, 3, 4552. 22077378 10.1021/am201352w

[16] M. Khosravi , S. Azizian , ACS Appl. Mater. Interfaces 2015, 7, 25326. 26496649 10.1021/acsami.5b07504

[17] A. J. Zhang , M. J. Chen , C. Du , H. Z. Guo , H. Bai , L. Li , ACS Appl. Mater. Interfaces 2013, 5, 10201. 24040904 10.1021/am4029203

[18] J. Li , L. Yan , X. Tang , H. Feng , D. Hu , F. Zha , Adv. Mater. Interfaces 2016, 3, 1500770.

[19] S. Liang , Y. Li , J. Yang , J. Zhang , C. He , Y. Liu , X. Zhou , Adv. Mater. Technol. 2016, 1, 1600117.

[20] W. Liu , Z. Chen , G. M. Zhou , Y. M. Sun , H. R. Lee , C. Liu , H. B. Yao , Z. N. Bao , Y. Cui , Adv. Mater. 2016, 28, 3578. 26992146 10.1002/adma.201505299

[21] S. Wang , S. H. Xuan , Y. P. Wang , C. H. Xu , Y. Mao , M. Liu , L. F. Bai , W. Q. Jiang , X. L. Gong , ACS Appl. Mater. Interfaces 2016, 8, 4946. 26835703 10.1021/acsami.5b12083

[22] H. B. Yao , J. Ge , C. F. Wang , X. Wang , W. Hu , Z. J. Zheng , Y. Ni , S. H. Yu , Adv. Mater. 2013, 25, 6692. 24027108 10.1002/adma.201303041

[23] S. B. Joye , Science 2015, 349, 592. 26250675 10.1126/science.aab4133

[24] M. Schrope , Nature 2011, 472, 152. 21490648 10.1038/472152a

[25] M. A. Shannon , P. W. Bohn , M. Elimelech , J. G. Georgiadis , B. J. Marinas , A. M. Mayes , Nature 2008, 452, 301. 18354474 10.1038/nature06599

[26] D. Chen , H. Zhu , S. Yang , N. Li , Q. Xu , H. Li , J. He , J. Lu , Adv. Mater. 2016, 28, 10443. 27781315 10.1002/adma.201601486

[27] I. Buist , S. Potter , T. Nedwed , J. Mullin , Cold Reg. Sci. Technol. 2011, 67, 3.

[28] D. X. Wu , Z. Y. Yu , W. J. Wu , L. L. Fang , H. T. Zhu , RSC Adv. 2014, 4, 53514.

[29] R. M. Atlas , T. C. Hazen , Environ. Sci. Technol. 2011, 45, 6709. 21699212 10.1021/es2013227 PMC3155281

[30] C. Y. Yan , Z. Y. Ji , S. H. Ma , X. L. Wang , F. Zhou , Adv. Mater. Interfaces 2016, 3, 1600015.

[31] J. Rubio , M. L. Souza , R. W. Smith , Miner. Eng. 2002, 15, 139.

[32] Q. L. Ma , H. F. Cheng , A. G. Fane , R. Wang , H. Zhang , Small 2016, 12, 2186. 27000640 10.1002/smll.201503685

[33] Z. X. Xue , Y. Z. Cao , N. Liu , L. Feng , L. Jiang , J. Mater. Chem. A 2014, 2, 2445.

[34] J. Ge , H.‐Y. Zhao , H.‐W. Zhu , J. Huang , L.‐A. Shi , S.‐H. Yu , Adv. Mater. 2016, 28, 10459. 27731513 10.1002/adma.201601812

[35] Z. L. Chu , Y. J. Feng , S. Seeger , Angew. Chem., Int. Ed. 2015, 54, 2328. 10.1002/anie.201405785 25425089

[36] L. P. Wen , Y. Tian , L. Jiang , Angew. Chem., Int. Ed. 2015, 54, 3387. 10.1002/anie.201409911 25614018

[37] H. G. Zhu , S. Yang , D. Y. Chen , N. J. Li , Q. F. Xu , H. Li , J. H. He , J. M. Lu , Adv. Mater. Interfaces 2016, 3, 1500683.

[38] L. B. Zhang , Z. H. Zhang , P. Wang , NPG Asia Mater. 2012, 4, e8.

[39] Y. Zhao , Z. Luo , M. H. Li , Q. Y. Qu , X. Ma , S. H. Yu , Y. L. Zhao , Angew. Chem., Int. Ed. 2015, 54, 919. 10.1002/anie.201408510 25422068

[40] W. B. Zhang , F. Liu , G. X. Liu , W. T. Gan , M. Zhang , H. Yu , X. Di , Y. Z. Wang , C. Y. Wang , Adv. Mater. Interfaces 2016, 3, 1600100.

[41] B. L. Xue , L. C. Gao , Y. P. Hou , Z. W. Liu , L. Jiang , Adv. Mater. 2013, 25, 273. 23074035 10.1002/adma.201202799

[42] Y. Voß , E. Wassel , S. Jiang , Q. Song , S. I. Druzhinin , H. Schönherr , Macromol. Biosci. 2016, DOI: 10.1002/mabi.201600337. 27762494

[43] E. Wassel , S. Y. Jiang , Q. M. Song , S. Vogt , G. Noll , S. I. Druzhinin , H. Schonherr , Langmuir 2016, 32, 9360. 27531168 10.1021/acs.langmuir.6b02708

[44] H. P. Cong , J. H. Qiu , S. H. Yu , Small 2015, 11, 1165. 25111389 10.1002/smll.201401651

[45] H. L. Che , M. Huo , L. Peng , T. Fang , N. Liu , L. Feng , Y. Wei , J. Y. Yuan , Angew. Chem., Int. Ed. 2015, 54, 8934. 10.1002/anie.201501034 26079643

[46] S. H. Ma , D. A. Wang , Y. M. Liang , B. Q. Sun , S. N. Gorb , F. Zhou , Small 2015, 11, 1131. 25331382 10.1002/smll.201402484

[47] T. Du , S. Ma , X. Pei , S. Wang , F. Zhou , Small 2017, 13, 160202. 10.1002/smll.201602020 27511623

[48] Q. Zhu , F. Tao , Q. M. Pan , ACS Appl. Mater. Interfaces 2010, 2, 3141. 20942429 10.1021/am1006194

[49] L. Zhang , L. L. Li , Z. M. Dang , J. Colloid Interface Sci. 2016, 463, 266. 26550784 10.1016/j.jcis.2015.10.065

[50] Y. Peng , Y. X. He , S. Yang , S. Ben , M. Y. Cao , K. Li , K. S. Liu , L. Jiang , Adv. Funct. Mater. 2015, 25, 5967.

[51] G. Kwon , A. K. Kota , Y. X. Li , A. Sohani , J. M. Mabry , A. Tuteja , Adv. Mater. 2012, 24, 3666. 22689385 10.1002/adma.201201364

[52] Y. Z. Cao , N. Liu , C. K. Fu , K. Li , L. Tao , L. Feng , Y. Wei , ACS Appl. Mater. Interfaces 2014, 6, 2026. 24397710 10.1021/am405089m

[53] M. M. Liu , J. Li , Z. G. Guo , J. Colloid Interface Sci. 2016, 467, 261. 26809105 10.1016/j.jcis.2016.01.024

[54] J. L. Song , Y. Lu , J. Luo , S. Huang , L. Wang , W. J. Xu , I. P. Parkin , Adv. Mater. Interfaces 2015, 2, 1500350.

