# Supplementary material for: Bioinspired, Mechano‐Regulated Interfaces for Rationally Designed, Dynamically Controlled Collection of Oil Spills from Water
Source: Glob Chall. 2017 Feb 15;1(3):1600014. doi: 10.1002/gch2.201600014 (PMC6607165; doi:10.1002/gch2.201600014)
Supplement: Supplementary file 1 — Supplementary [file GCH2-1-1600014-s001.pdf]

Copyright WILEY-VCH Verlag GmbH & Co. KGaA, 69469 Weinheim, Germany, 2017.

Global Challenges
Open Access

Supporting Information

for *Global Challenges,* DOI: 10.1002/gch2.201600014

Bioinspired, Mechano-Regulated Interfaces for Rationally Designed, Dynamically Controlled Collection of Oil Spills from Water

*Yaoyao Li, Deyong Zhu, Stephan Handschuh-Wang, Guanghui Lv, Jiahui Wang, Tianzhen Li, Cancheng Chen, Chuanxin He, Junmin Zhang, Yizhen Liu, Bo Yang, and Xuechang Zhou**

Copyright WILEY-VCH Verlag GmbH & Co. KGaA, 69469 Weinheim, Germany, 2016.

Supporting Information

Bio-Inspired, Mechano-Regulated Interfaces for Rationally-Designed, Dynamically-Controlled Collection of Oil Spills from Water

*Yaoyao Li, Deyong Zhu, Stephan Handschuh-Wang, Guanghui Lv, Jiahui Wang, Tianzhen Li, Cancheng Chen, Chuanxin He, Junmin Zhang, Yizhen Liu, Bo Yang, and Xuechang Zhou**

College of Chemistry and Environmental Engineering,

Shenzhen University,

Shenzhen, 518060, P. R. China

E-mail: xczhou@szu.edu.cn (X. C. Z.)

Experimental Section

Fabrication of the Ecoflex sponges: The sugar cubes were immersed into the Ecoflex liquid prepolymer (solution A and solution B, 1:1) in a plastic cup. Afterwards, the prepolymer was degassed in a vacuum chamber for 10 minutes. During the degassing the liquid Ecoflex prepolymer infiltrated the voids of the sugar cubes. Thereafter, the sugar cubes with the absorbed mixture were cured at 65 °C for 40 minutes in a petri dish. Subsequently, the sugar was dissolved in water and washed away at 65 ^{o}C under stirring. Then the sample was cleaned with ethanol. Finally, Ecoflex sponges were obtained after drying at 65 ^{o}C at atmospheric pressure for 2~3 h.

Characterization of the Ecoflex sponge: The morphology was determined with a scanning electron microscope (S-3400N, Hitachi) at an acceleration voltage of 15 keV. The elastic modulus was measured with a digital tensile testing machine (CMT4304, Suns). The wettability of the sponge was determined by contact angle measurement with a computer based contact angle microscope (SDC-200, Sindin). A commercially available dye (Oil red O, Macklin) was used to better discern oil phase and water phase in an oil/water mixture.

Characterization of the absorption capacity of Ecoflex sponges: The absorption capacity of the Ecoflex sponge was measured for various organic solvents and oils, including ethanol (0.79 gcm^{-3}, 99.7%, Aladdin), methanol (0.79 gcm^{-3}, 99.9%, McClean), isopropanol (0.78

gcm^{-3}, 99.5%, McClean), acetone (0.79 gcm^{-3}, 99.5%, Beijing SHIJI), paraffin oil (0.88 gcm^{-3}, 99.0%, McClean), hexane (0.66 gcm^{-3}, 97.0%, Aladdin), toluene (0.87 gcm^{-3}, 99.5%, McClean) dicholoromethane (1.33 gcm^{-3}, 99.8%, McClean), trichloromethane (1.48 gcm^{-3}, 99.8%, McClean), FC-40 (1.85 gcm^{-3}, 3M), and peanut oil (0.92 gcm^{-3}, Wilmar). The absorption capacity of the sponge is dependent on the specific liquid. The experiment was done by dipping the sponge into an organic liquid and subsequent releasing the liquid via squeezing. The sponge weight before release (m_{sp1}) and after release of the organic liquid (m_{sp2}) was recorded. The absorption capacity (c) was calculated by Equation 1.

$$c = \frac{(m_{sp1} - m_{sp2})}{m_{sp2}} \cdot 100\% \qquad (1)$$

Characterization of the oil flux of Ecoflex sponges:

The weight of the filled skimmer (m_{F}) was measured and afterwards, the organic liquid was poured out via the sponge lid, while the time (t) for pouring out the liquid was measured, and finally the weight of the empty glass vial (m_{E}) was determined. The oil flux (J) was calculated by Equation 2 utilizing the surface area (A) of the lid made of the polymeric sponge.

$$J = \frac{m_F - m_E}{t \cdot A} \qquad (2)$$

Movie 1: Ecoflex sponges exhibit an ultra-high flexibility which is rarely observed in other highly porous materials (e.g., PDMS sponge and PU sponge). Manual compression with a volume reduction of over 80% and an elongation at break of nearly 700% show that the ecoflex sponges are highly flexible and springy.

Movie 2: When paraffin oil was dropped onto the surface of the ecoflex sponge or onto the surface of water beside the ecoflex sponge, the oil was quickly and automatically absorbed into the sponge.

Movie 3: Several small cubes of ecoflex sponges were added into a beaker containing the paraffin oil/water mixture. The beaker was sealed with Parafilm and next shaken by hand to thoroughly mix the liquids and the ecoflex sponges. The results revealed that the paraffin oil on the water surface was completely absorbed by the ecoflex sponges, and the separation of oil/water mixture was achieved simply by pouring the remained water into another beaker.

Movie 4: Several small cubes of ecoflex sponges were added into a beaker containing a mixture of water and chloroform (labeled with Oil red O dye). After vigorous stirring, the red-doped chloroform was totally absorbed by the sponges. Finally, water was recovered from the chloroform/water mixture by simply pouring out through the punched holes.

Movie 5: 50 mL of paraffin oil labeled with Oil red O dye was added in a beaker while floating on the water surface. Then an oil skimmer covered with a piece of ecoflex sponge was placed on the surface of the oil/water mixture. It was found that the oil skimmer floated at the surface without sinking. Due to the superhydrophobicity and superoleophilicity of ecoflex sponges, the upper paraffin oil, yet no water, went into the oil skimmer, which led to that the oil skimmer tilted gradually because of the gravity center shift. Within a few minutes, the oil skimmer became vertical to the water surface and stopped absorbing paraffin oil.

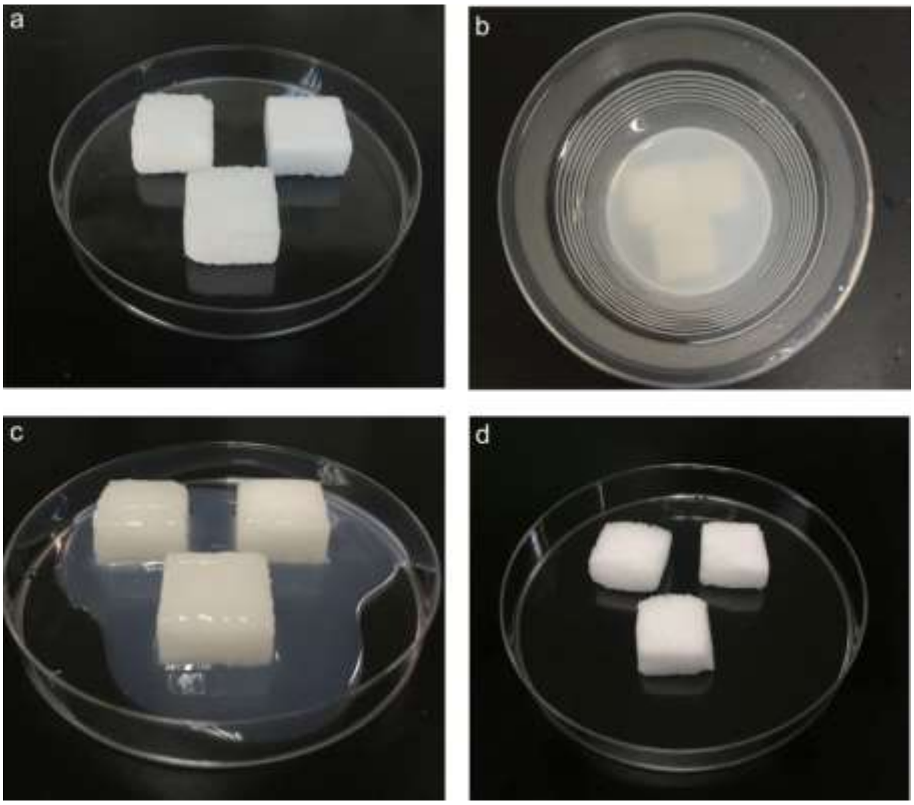

Figure S1. (a) Photograph of sugar cubes. (b) Loading of the sugar cubes with the Ecoflex pre-polymer in a plastic beaker. (c) Cured Ecoflex-sugar composite (d) As-made Ecoflex sponges.

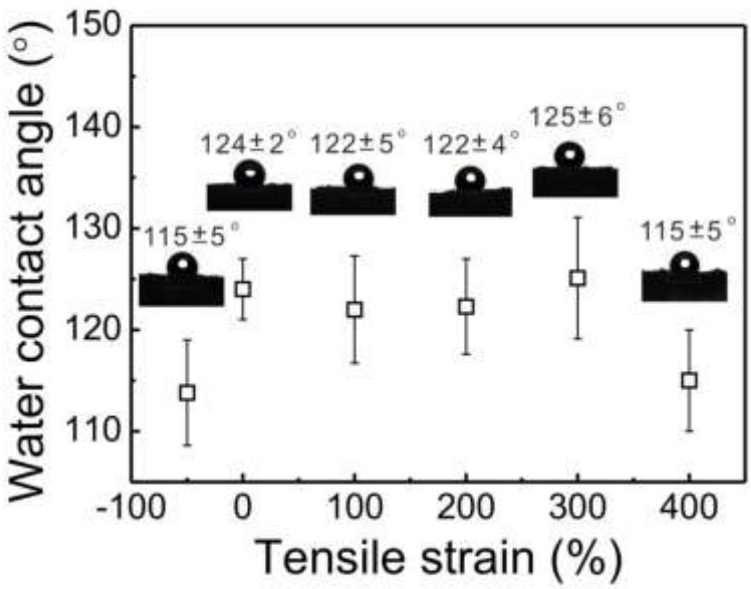

Figure S2. Water contact angle of the as-made ecoflex sponges at different tensile strain increasing from -50%, 0, 100%, 200%, 300%, and 400%.

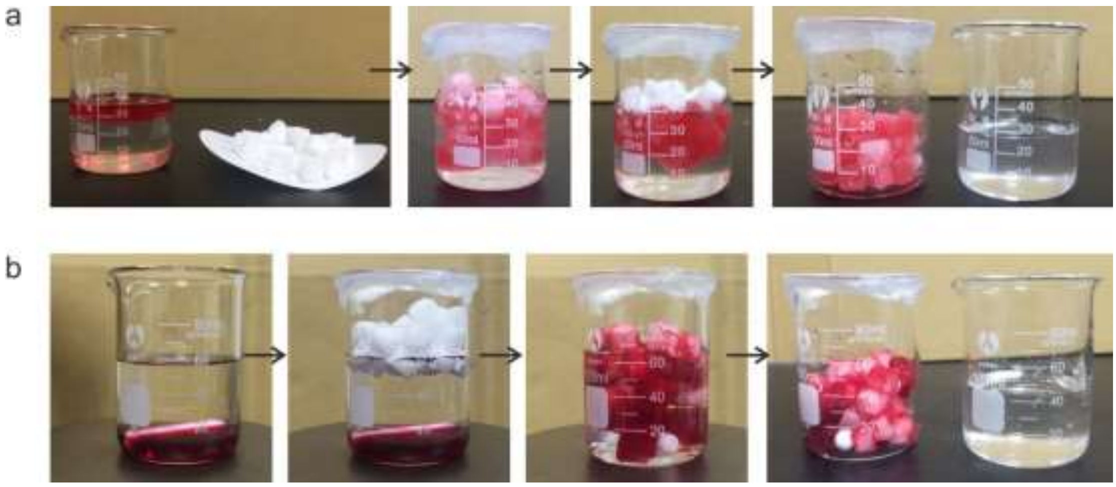

Figure S3. Optical graphs show the separation of water/paraffin oil (a) and water/chloroform (b) with the as-made ecoflex sponges, respectively. Both of the paraffin oil and chloroform were indicated by doping with a oil-soluble red dye.

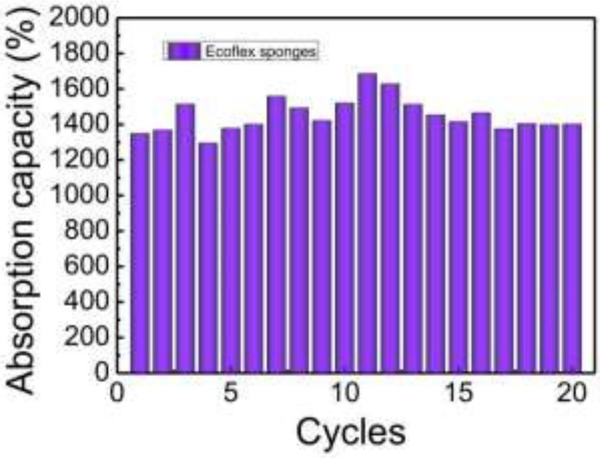

Figure S4. Absorption capacity of chloroform at different loading cycles.
